# Hyperconnectivity and altered interactions of a nucleus accumbens network in post-stroke depression

**DOI:** 10.1093/braincomms/fcac281

**Published:** 2022-11-02

**Authors:** Lena K L Oestreich, Paul Wright, Michael J O’Sullivan

**Affiliations:** UQ Centre for Clinical Research, The University of Queensland, Brisbane 4072, Australia; Centre for Advanced Imaging, The University of Queensland, Brisbane 4072, Australia; Biomedical Engineering Department, King’s College London, London, UK; UQ Centre for Clinical Research, The University of Queensland, Brisbane 4072, Australia; Biomedical Engineering Department, King’s College London, London, UK; Department of Neurology, Royal Brisbane and Women’s Hospital, Brisbane 4072, Australia; Institute of Molecular Bioscience, The University of Queensland, Brisbane 4072, Australia

**Keywords:** resting-state functional MRI, diffusion MRI, post-stroke depression, stroke, default mode network (DMN)

## Abstract

Post-stroke depression is a common complication of stroke. To date, no consistent locus of injury is associated with this complication. Here, we probed network dynamics and structural alterations in post-stroke depression in four functional circuits linked to major depressive disorder and a visual network, which served as a control network. Forty-four participants with recent stroke (mean age = 69.03, standard deviation age = 8.59, age range = 51–86 and gender: female = 10) and 16 healthy volunteers (mean age = 71.53, standard deviation age = 10.62, age range = 51–84 and gender: female = 11) were imaged with 3-Tesla structural, diffusion and resting-state functional MRI. The Geriatric Depression Scale was administered to measure depression severity. Associations between depression severity and functional connectivity were investigated within networks seeded from nucleus accumbens, amygdala, dorsolateral prefrontal cortex and primary visual cortex. In addition, the default mode network was identified by connectivity with medial prefrontal cortex and posterior cingulate cortex. Circuits that exhibited altered activity associated with depression severity were further investigated by extracting within-network volumetric and microstructural measures from structural images. In the stroke group, functional connectivity within the nucleus accumbens-seeded network (left hemisphere: *P* = 0.001; and right hemisphere: *P* = 0.004) and default mode network (cluster one: *P* < 0.001; and cluster two: *P* < 0.001) correlated positively with depressive symptoms. Normal anticorrelations between these two networks were absent in patients with post-stroke depression. Grey matter volume of the right posterior cingulate cortex (Pearson correlation coefficient = −0.286, *P* = 0.03), as well as microstructural measures in the posterior cingulate cortex (right: Pearson correlation coefficient = 0.4, *P* = 0.024; and left: Pearson correlation coefficient = 0.3, *P* = 0.048), right medial prefrontal cortex (Pearson correlation coefficient = 0.312, *P* = 0.039) and the medial forebrain bundle (Pearson correlation coefficient = 0.450, *P* = 0.003), a major projection pathway interconnecting the nucleus accumbens-seeded network and linking to medial prefrontal cortex, were associated with depression severity. Depression after stroke is marked by reduced mutual inhibition between functional circuits involving nucleus accumbens and default mode network as well as volumetric and microstructural changes within these networks. Aberrant network dynamics present in patients with post-stroke depression are therefore likely to be influenced by secondary, pervasive alterations in grey and white matter, remote from the site of injury.

## Teaser

Oestreich et al. (2022) report reduced mutual inhibition between functional circuits involving nucleus accumbens and default mode network in stroke patients suffering from depression. The use of advanced multimodal imaging approaches revealed that these functional aberrations were accompanied by volumetric and microstructural changes within these networks.

## Graphical Abstract

**Graphical abstract:**
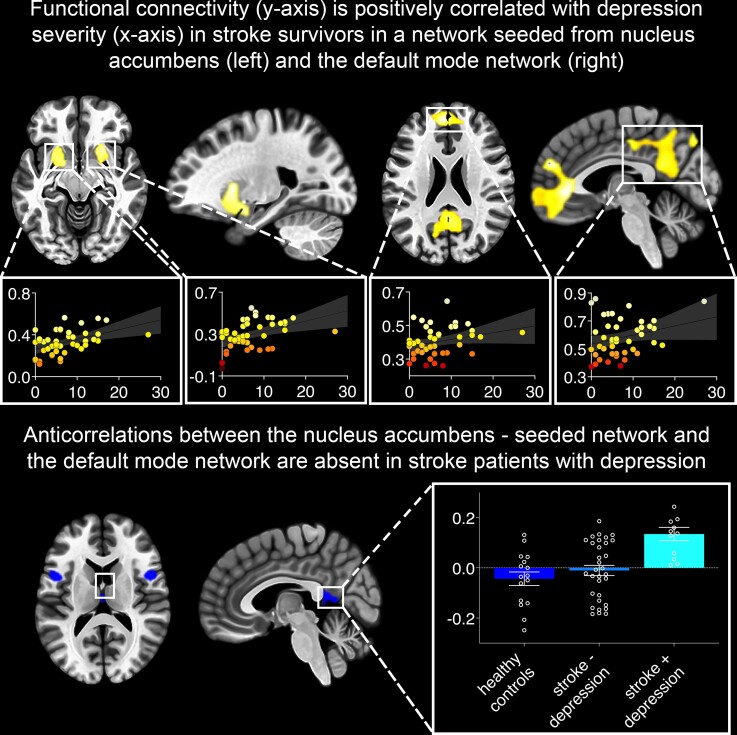

## Introduction

For approximately one-third of stroke survivors, rehabilitation outcomes are adversely affected by post-stroke depression (PSD).^1^ PSD is partly driven by mechanisms specific to brain injury rather than the psychological reaction to physical disability alone: stroke survivors exhibit higher rates of depression than physically ill patients with similar levels of disability.^2^ However, systematic reviews^3^ and meta-analyses^4^ have found no simple relationship between lesion size or location and PSD. One possible explanation is that infarction initiates a cascade of events, whereby alterations in brain structure and function propagate away from the site of injury so that key alterations critical to behaviour come to be present outside the extent of visible injury on standard MRI. Advances in microstructural and functional MRI provide a methodology to characterize such alterations.

Studies in animal models of depression and major depressive disorder (MDD) in the absence of injury identify candidate networks that could also be implicated in the development of depression after stroke. The amygdala receives sensory input from thalamic projections^5^ and facilitates affective regulation in response to sensory stimuli via connections to dorsal anterior cingulate cortex (dACC) and insula.^6^ In MDD, hyperconnectivity accompanied by enlargement of amygdala has been observed.^7^ The nucleus accumbens (NAc) and associated networks are implicated by animal models of depression: the NAc receives dopaminergic input from the ventral tegmental area via the medial forebrain bundle, a major projection tract, that carries the mesolimbic dopamine pathway.^8^ Experimental inhibition of mesolimbic dopamine release leads to impaired defensive reactions during adverse conditions (learned helplessness) and blunted responses to reward (anhedonia),^9^ two cardinal features of depression in humans. The NAc modulates reward processing by integrating inputs from limbic and cortical regions including hippocampus, anterior cingulate and orbitofrontal cortex^10^ and also receives efferent projections from the amygdala.

Large-scale cortex-embedded networks have also been implicated in depressive symptoms in MDD, potentially via reduced top-down control of emotion processing.^11^ Limited responsiveness^12^ and smaller volumes^13^ of dorsolateral prefrontal cortex have been reported in MDD. Attenuated functional connectivity in this network has been observed 10 days^14^ and 3 months^15^ post-stroke. Another feature of studies in MDD is altered dynamic interaction between networks, for example, between the salience network and DMN, leading to synchronous activation of both networks, which has been linked to difficulties for depressed patients to shift from self-focused thinking to goal-directed behaviour.^16^ This type of reciprocal network interaction has not been investigated in stroke. One advantage of doing so is that the discovery of altered network dynamics, coinciding with emergence of depression, would provide stronger causal evidence for the role of network interactions in the development of depressive symptoms.

Based on the previous literature in animal models of depression and MDD, the primary aim of this study was to investigate whether functional networks typically affected in MDD without brain injury also exhibit aberrant neural dynamics in individuals who experience depression after stroke. We further probed cross-network dynamics of these networks via correlation of activity with the DMN. A secondary aim was to explore mechanistic underpinnings of network dynamics associated with PSD. For this purpose, we conducted follow-up analyses in circuits exhibiting depression-related activity by testing properties of grey matter regions and white matter connections that provide their structural architecture.

## Materials and methods

### Participants

A sample of 46 patients with first ischemic stroke was recruited from a larger cohort enrolled into a longitudinal study of cognitive outcome after stroke (STRATEGIC, NCT03982147). Participants were aged over 50 years and had a diagnosis of ischemic stroke confirmed by neuroimaging. Exclusion criteria were dementia, previous stroke, major neurological disease, history of psychiatric disorder based on DSM-IV criteria, including major depressive disorder, previous moderate to severe head injury, inability to converse fluently in English, active malignancy and any other factors that would affect performance of cognitive tasks (e.g. aphasia, visual impairment etc.). Participants also completed the Geriatric Depression Scale (GDS), a 30-item self-report measure designed to identify depression in older people.^17^ Sixteen age- and handedness-matched healthy controls (HC) were recruited from the community. The study protocol was approved by the London and Bromley Research Ethics Committee (REC reference: 13-LO-1745) and the University of Queensland Research Ethics Committee (Clearance number: 2018001541). All participants gave written informed consent. Two participants were excluded due to motion artefacts in the resting-state functional MRI (rs-fMRI) scans.

### MRI acquisition and processing

Research MRI was acquired on a 3T MR750 MR scanner (GE Healthcare, Little Chalfont, Buckinghamshire, United Kingdom), within 30–95 days of stroke (*M* = 69.7, *SD* = 17.4). T1-weighted imaging was performed with the magnetization-prepared rapid acquisition gradient echo (MPRAGE) sequence.^18^ Diffusion-weighted images were acquired with an echo-planar imaging (EPI) sequence using 60 diffusion-sensitization directions at *b* = 1500 s/mm^2^ and six acquisitions without diffusion sensitization (*b* = 0). T2-weighted fluid-attenuated inversion recovery (FLAIR) and fast recovery fast spin echo (FRFSE) sequences were acquired for lesion delineation. Detailed acquisition parameters for these structural sequences have been published previously.^19^ Rs-fMRI was acquired with an echo-planar imaging sequence using the following parameters: TR = 2000 ms, TE = 30 ms, flip angle = 75°, slice thickness = 3 mm and gap = 0.3 mm. Images were acquired in the axial plane with FOV = 211 × 211 mm^2^, matrix size = 64 × 64 mm^2^ and number of slices = 40. A total of 180 volumes were collected over a scan time of 7 min. Participants were instructed to keep their gaze on a fixation cross throughout the scan.

### Rs-fMRI processing

Data were pre-processed and analysed using the functional connectivity toolbox CONN version 19b (www.nitrc.org/projects/conn, RRID:SCR_009550),^20^ implemented in MATLAB R2020a and SPM12 (https://www.fil.ion.ucl.ac.uk/spm/software/spm12/). The first 10 volumes of each participant’s fMRI data were discarded to allow for magnetization equilibrium. After brain extraction, the T1-weighted images were centred (coordinates = 0, 0, 0), segmented and normalized. Images were smoothed with an 8 mm fullwidth at half-maximum isotropic Gaussian kernel. Denoising was performed by utilizing the anatomical component-based noise correction procedure (CompCor), regressing noise components from cerebrospinal fluid and cerebral white matter,^21^ outlier scans or scrubbing identified during pre-processing,^22^ estimated subject-motion parameters,^23^ as well as constant and first-order linear session effects^20^ out of the fMRI time series. The data were then temporally bandpass-filtered (0.01–0.08 Hz) to remove functional images with linear trends.

Functional connectivity (FC) networks were defined based on seed regions in the nucleus accumbens (NAc-seeded network), amygdala (amygdala-seeded network) and dorsolateral prefrontal cortex (dlPFC-seeded network). The DMN was reconstructed by using seed regions in posterior cingulate cortex (PCC) and medial prefrontal cortex (mPFC). A visual network, seeded from the primary visual cortex (V1-seeded network), was used as a control network. Seeds were defined as spheres with a radius of 6 mm (see Supplementary Fig. 1), placed at the MNI coordinates of the NAc (left: −12, 08, −12 and right: 10, 10, −12), amygdala (left: −24, 0, −21 and right: 21, −1, −22), dlPFC (left: −46, 38, 8 and right: 43, 38, 12), PCC (left: −39, 34, 37 and right: 35, 39, 31), mPFC (left: −5, 17, −13 and right: 7, 17, −14) and V1 (left: −11, −81, 7 and right: 11, −78, 9). Individual FC maps were generated for each seed region, using averaged time series. The FC strength between seed region and all brain voxels was quantified by the Pearson’s cross-correlation. Fisher’s r-to-z transformation was applied to improve the normal distribution of the correlation coefficients. All analyses were FWE-corrected at a cluster threshold of *P* < 0.05.

### T1/DWI processing

T1-weighted images were preprocessed with the *recon-all* command implemented in FreeSurfer (v6.0) (http://surfer.nmr.mgh.harvard.edu/) to reconstruct cortical and subcortical parcellations based on the Desikan-Killiany atlas.^24^ Parcellations were validated by manual inspection. The diffusion-weighted data were corrected for head movements, eddy current distortions and magnetic field inhomogeneities, using tools implemented in MRtrix3.^25^ A five-tissue-type segmented image, suitable for use of anatomically constrained tractography, was generated from the pre-processed T1-weighted images. Free-water imaging was used to correct for CSF contamination and to quantify the amount of extracellular free-water (FW) by separating the diffusion properties of brain tissue from the surrounding extracellular free-water.^26^ Response functions were estimated using the single-fibre *tournier* algorithm^27^ and constrained spherical deconvolution (CSD) was applied to obtain fibre orientation distributions (FOD). Anatomically constrained tractography with the second order integration over Fibre Orientation Distribution (iFOD2) algorithm^25^ was used to generate individual tractograms for each participant.^28^ Tractograms were generated until 100 million streamlines were obtained with a length of 5–250 mm, step size of 1 mm and FOD amplitude threshold of 0.1. The spherical deconvolution informed filtering of tractograms (SIFT) algorithm was applied to reduce the overall streamline count to 10 million, providing more biologically meaningful estimates.^29^

#### Follow-up analysis of volume and microstructure in functional networks associated with PSD

Volumes and extracellular FW estimates were generated for relevant brain structures generated from FreeSurfer parcellations, namely, NAc, mPFC and PCC. Clusters with significant FC to seed regions associated with GDS scores in any of the five networks were extracted as binary masks and registered to individual subject space. These masks together with the original seed regions were then used as inclusion regions of interest (ROIs) in global tractography through which white matter tracts had to pass to be maintained from the original whole-brain tractograms. Free-water corrected (tissue-specific) fractional anisotropy (FA_t_) and FW were extracted and averaged across each tract. Fibres in the DMN could not be reconstructed for three participants from the group of stroke patients without depression.

### Lesion definition and overlap with networks

FLAIR images were used to draw lesions manually. When necessary, acutely acquired diffusion images were used to identify the infarct. Lesion maps and white matter tracts were co-registered together with the rs-fMRI networks into MNI standard space so that anatomically homologous brain areas were aligned.

### Statistical analysis

While most analyses were performed using GDS scores as a continuous variable, patients with stroke were split into groups of patients with PSD (D+) and patients free of PSD (D−) for some analyses to facilitate comparisons to the HC group. Stroke groups were split based on a score of 10 on the GDS, which has previously been identified to yield the highest sensitivity (0.69) and specificity (0.75) in stroke samples.^30^ Thirty-two (72.7%) stroke patients had a score below 10 and 12 (27.3%) patients scored 10 or above on the GDS and were assigned to the D− and D+groups, respectively. Previous studies have suggested that apathy and depression are dissociable post-stroke syndromes with different effects on outcome after stroke.^31^ Additional analyses with apathy-items from the GDS are therefore provided in the Supplementary. To investigate whether FC in any of the five networks was associated with PSD, GDS scores of participants in the stroke group were regressed onto the whole-brain FC maps estimated from each of the seed regions. Average FC was then extracted from clusters that were significantly associated with GDS scores, individually for each participant. A repeated-measures analysis of covariances (ANCOVA) with *group* (HC/D+/D−) as between-subjects factor and *network* and *cluster* as within-subjects factors was calculated to determine if FC in these clusters varied across groups. Several regions of the identified resting-state networks are anticorrelated with one another (see Supplementary Fig. 1). To investigate whether FC changes associated with PSD are driven by insufficient deactivation of anticorrelated networks, a multivariate analysis of covariances (MANCOVA) with *grou*p as between-subjects factor and average *FC* (extracted from anticorrelated clusters) as within-subjects factor was performed.

#### Follow-up statistical analysis of volume and microstructure in functional networks associated with PSD

Two repeated-measures ANCOVA with *group* as between-subjects factor, as well as *volume* or *FW* and *hemisphere* as within-subjects factors, were conducted to test for structural alterations in the seed regions between groups. To determine whether BOLD time-series correlations between seed regions and clusters associated with PSD were accompanied by underlying changes in white matter microstructure, Pearson’s correlation coefficients between GDS scores and microstructural measures (FA_t_/FW) were calculated in the stroke sample only. Group differences were tested using a MANCOVA with the between-subjects factor *group* and the within-subjects factor *white matter estimates*.

Overlap of resting-state networks, grey matter regions and white matter tracts with lesions were quantified with the Jaccard index, which was compared between groups and tested for associations with GDS scores.

Age and sex were added as covariates to all analyses. *P*-values were adjusted using the Bonferroni correction of *P* = α/*k,* whereby alpha was set to 0.05 and k denoted the number of comparisons. Due to the small sample size of this study, t-tests used to test associations between GDS scores and FC in the five resting-state networks, as well as all Pearson correlation coefficients, were additionally presented with bootstrapped confidence intervals (CI) of the t-statistic and correlation coefficient, respectively. Bootstrap resampling (1000 iterations) was used to assess the reliability of brain-depression associations by constructing bias-corrected and accelerated 95% confidence intervals (BCa 95% CI).

### Data availability

The data presented in this study will be made available upon request. Requests should be directed to the chief investigator (MJO), as defined in the study protocol.

## Results

Stroke and HC groups did not differ in age, handedness or smoking status, but there were more men in the stroke group than in the HC group (see Table 1). The D+ and D− groups had a similar gender ratio of approximately 1:3 women to men [χ(1) = 0.05, *P* = 0.826]. Due to the difference in sex ratio between groups, all main analyses were repeated without sex as a covariate and are presented in the Supplements.

**Table 1:** Demographics and lesion characteristics by group

Variable	HC (*n* = 16)	D+ (*n* = 12)	D− (*n* = 32)	Group comparisons
	Mean (*SD*)/category	Mean (*SD*)/category	Mean (*SD*)/category	
*Demographics*				
Age (years)	71.53 (10.62)	69.72 (6.96)	68.78 (9.22)	*F*(2,57) = 0.48, *P* = 0.62
Sex (female/male)	11/5	3/9	7/25	χ^2^(2) = 10.96, *P* = 0.004
Handedness (right/left)	16/0	11/1	31/1	χ^2^(2) = 1.49, *P* = 0.48
Smoking status (never/previously/current)	10/5/1	5/6/1	18/9/5	χ^2^(4) = 2.79, *P* = 0.59
*Lesion characteristics*				
Hemisphere (left/right/bilateral)		5/7/0	18/13/1	χ^2^(2) = 1.33, *P* = 0.51
Arterial territory (ACA/MCA_ant_/MCA_pos_/MCA_str_/PCA/lacunar/thalamic)		0/2/3/3/2/1/1	1/7/7/4/6/5/2	χ^2^(6) = 1.80, *P* = 0.94
Volume (ml)		7175.64 (12081.03)	7600.81 (11962.09)	*t*(42) = 0.01, *P* = 0.92
Time since lesion (days)		68.17 (13.39)	70.28 (18.85)	*t*(42) = 0.13, *P* = 0.72

HC = healthy controls; D + = Post-stroke depression group; D− = stroke group free of depression; SD = standard deviation; MCA_ant_ = middle cerebral artery, anterior; ACA = anterior cerebral artery; MCA_pos_ = middle cerebral artery, posterior; MCA_str_ = middle cerebral artery, striatocapsular; PCA = posterior cerebral artery.

### Resting-state networks

The five seeded FC networks averaged across all participants and their locations are shown in Supplementary Fig. 1 and their cluster size and locations are provided in Supplementary Table 1. Briefly, a positive BOLD time-series correlation was found between NAc and a single large cluster confined to adjacent subcortical structures but extending to insula and medial frontal cortex (including parts of the dorsal and ventral ACC as well as orbitofrontal cortex). The amygdala-seeded network subsumed similar subcortical structures and medial frontal regions but also involved parts of the temporal lobe (parahippocampal gyrus, middle temporal gyrus, superior temporal gyrus, temporal pole) and lingual gyrus. The dlPFC-seeded network included bilateral clusters including the frontal pole, inferior frontal gyrus and superior frontal gyrus, bilateral clusters spanning across regions in the lateral occipital cortex, angular gyrus and supramarginal gyrus and a medial cluster including dorsal ACC and superior frontal gyri. The DMN and V1-seeded networks corresponded with the typical configuration of these networks described in previous literature (Supplementary Fig. 1). Supplementary Fig. 1 also shows regions anticorrelated with each of these networks. Of note, BOLD activity in the NAc network was anticorrelated with a region of PCC that was part of a larger region within the DMN.

### Associations between PSD and resting-state networks

**Figure 1:**
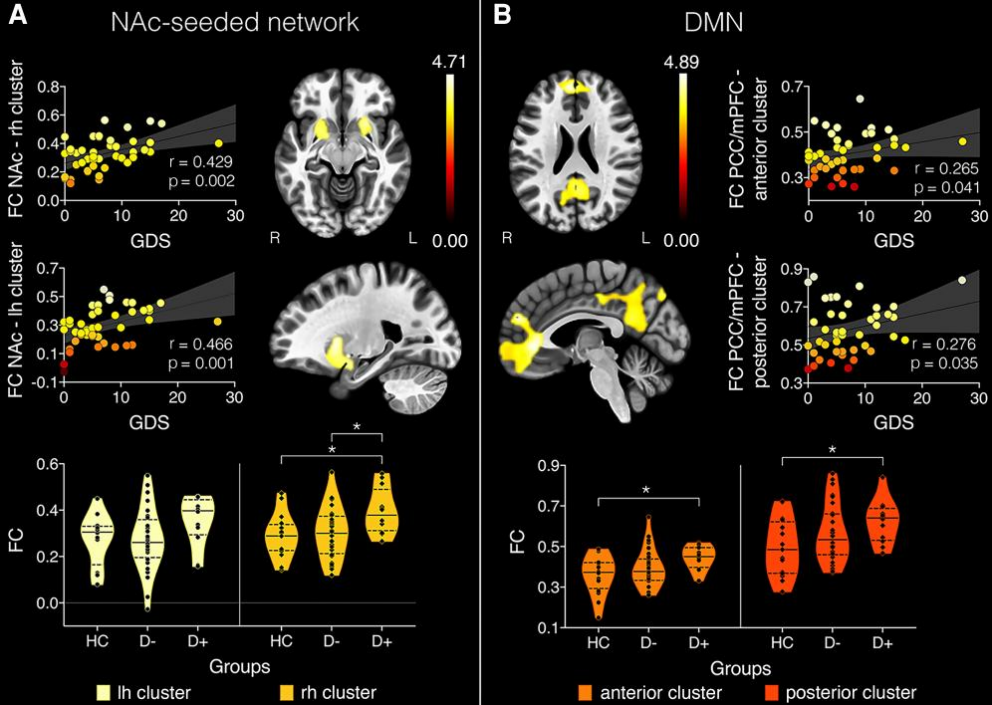
**Clusters with significant BOLD time-series correlations with geriatric depression scale (GDS).** (**A**) in nucleus accumbens (NAc)-seeded network and (**B**) the default mode network (DMN). Colorbars represent t-statistic values and data points in scatterplots are color-coded according to their corresponding t-statistic. Pearson correlations were used to calculate correlation coefficients (r) and *P*-values (*P*). Violin plots show the frequency distribution of the data, including the median (continuous line) and quartiles (dashed line). FC = functional connectivity; PCC = posterior cingulate cortex; mPFC = medial prefrontal cortex; rh = right hemisphere; lh = left hemisphere; R = right; L = left; HC = healthy controls; D− = stroke patients free of depression and D+ = patients with post-stroke depression. Violin plots represent the distributions of FC values per group by cluster. *P < 0.05.

Within the stroke group, GDS scores were associated with BOLD signal correlations between NAc and one cluster in the left (*t*(43) = 4.65, *P* = 0.001, *k* = 792, BCa 95% CI [0.24, 0.66]) and one in the right (*t*(43) = 4.64, *P* = 0.004, *k* = 607, BCa 95% CI [0.22, 0.67]) hemisphere (see Fig. 1). Both clusters included the putamen, amygdala, orbitofrontal cortex and insula. The cluster seeded from the right NAc additionally covered areas of the pallidum and hippocampus in the right hemisphere. Within the DMN, GDS scores were associated with BOLD time-course correlations between PCC and a cluster including the medial frontal cortex and frontal poles [*t*(43) = 6.76, *P* < 0.001, *k* = 3789, BCa 95% CI [0.09, 0.57]]. GDS scores were furthermore associated with BOLD time-course correlations between the mPFC and a cluster in the precuneus and PCC [*t*(43) = 5.56, *P* < 0.001, *k* = 3251, BCa 95% CI [0.04, 0.47]]. In all four identified clusters, GDS increased as FC increased, indicating that hyperconnectivity within the NAc-seeded network and the DMN are associated with depression severity in stroke patients. No associations between GDS scores and FC in the amygdala-, dlPFC or V1-seeded networks were observed.

Average FC was extracted from each of the four clusters associated with GDS scores individually for each participant. A repeated-measures ANCOVA identified a significant main effect of *group* [*F*(2,55) = 6.25, *P* = 0.004, *n_p_^2^* = 0.183]. As can be seen in Fig. 1, Bonferroni corrected post-hoc tests (12 comparisons) found that FC between the right hemisphere cluster and NAc was significantly increased in the D+ group compared to the HC [*t*(26) = 2.70, *P* = 0.027] and D− [*t*(42) = 2.68, *P* = 0.029] groups. Furthermore, FC in clusters correlated with the DMN seeds was significantly increased in the D+ group compared to the HC group [anterior cluster: *t*(26) = 2.52, *P* = 0.044; posterior cluster: *t*(26) = 2.51, *P* = 0.045]. No significant differences in FC were observed between the D+ and D− groups.

### Anticorrelations between DMN and NAc-seeded network

**Figure 2:**
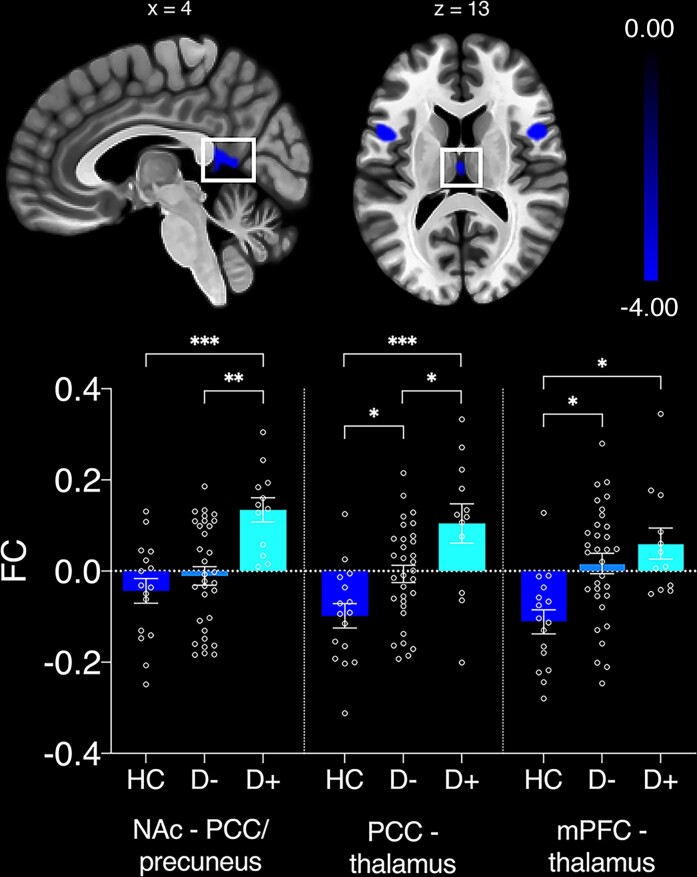
**Significant group differences in anticorrelated clusters of nucleus accumbens (NAc)-seeded network and default mode network (DMN).** Group differences are based on Bonferroni-corrected post-hoc tests. FC = functional connectivity; PCC = posterior cingulate cortex and mPFC = medial prefrontal cortex. Error bars represent standard errors of the mean. *P < 0.05; **P < 0.01; and ***P < 0.001.

Only the NAc-seeded network exhibited significant anticorrelations with regions in the DMN. For every participant, average FC was extracted from anticorrelated clusters. A MANCOVA identified main effects of *group* for FC between bilateral NAc and PCC/precuneus [*F*(4,55) = 11.8, *P* < 0.001, *n_p_^2^* = 0.293], PCC and thalamus [*F*(4,55) = 10.44, *P* < 0.001, *n_p_^2^* = 0.268] and mPFC and thalamus [*F*(4,55) = 4.75, *P* = 0.012, *n_p_^2^* = 0.143] (see Fig. 2). Specifically, Bonferroni corrected post-hoc tests (12 comparisons) identified significantly decreased FC (i.e. a greater degree of anticorrelation) in the HC group compared to the D+ group in all three clusters [bilateral NAc—PCC/precuneus: *t*(26) = 4.68, *P* < 0.001; PCC—thalamus: *t*(26) = 4.57, *P* < 0.001; mPFC—thalamus: *t*(26) = 2.86, *P* = 0.018]. The D+ group also exhibited reduced anticorrelated FC compared to the D− group between the bilateral NAc and PCC/precuneus [*t*(42) = 4.01, *P* = 0.001] and PCC and thalamus [*t*(42) = 2.81, *P* = 0.02] clusters. Lastly, the D− group had significantly increased FC compared to the HC between PCC and thalamus [*t*(46) = 2.59, *P* = 0.037] and mPFC and thalamus [*t*(46) = 2.5, *P* = 0.046]. As can be seen in Fig. 2, on average, FC values changed from negative to positive in the D+ group for all three clusters, indicating that DMN activity in these areas is not adequately suppressed during activation of the NAc-seeded network and vice versa.

#### Follow-up analysis of volume and microstructure in functional networks associated with PSD: NAc-seeded network and DMN

##### Grey matter volume and microstructure

**Figure 3:**
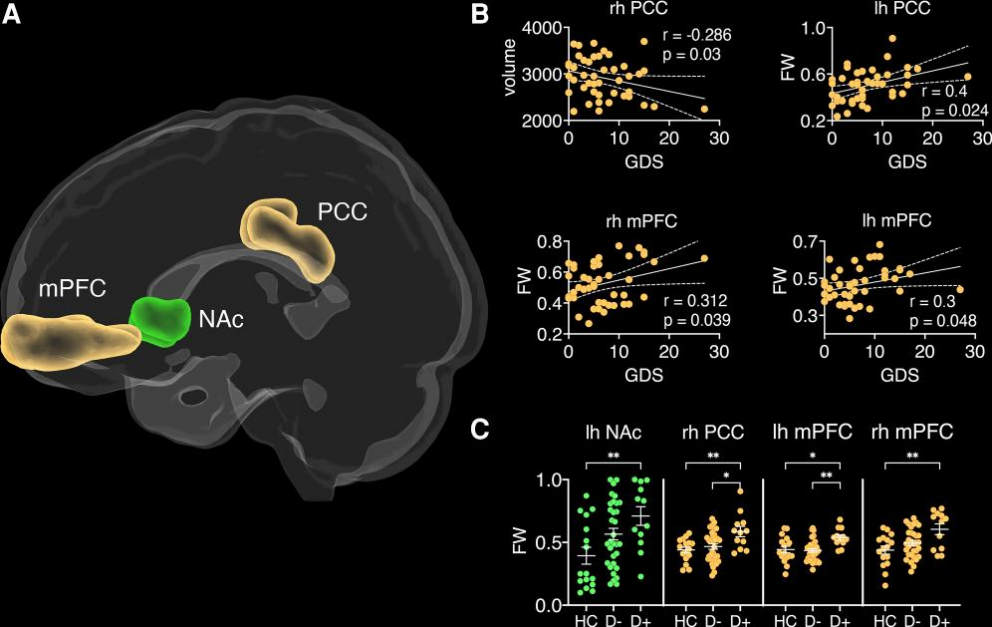
**Grey matter seed regions of resting-state networks.** (**A**) PCC = posterior cingulate cortex (yellow), mPFC = medial prefrontal cortex (yellow), NAc = nucleus accumbens (green). **(B)** Correlations between geriatric depression scale (GDS) scores and free-water (FW)/volume of grey matter regions. Error bands represent standard error, rh = right hemisphere and lh = left hemisphere. **(C)** Group differences in FW for different grey matter structures based on Bonferroni-corrected post-hoc tests. HC = healthy controls, D + = stroke patients with depression and D− = stroke patients free of depression. *P < 0.05; **P < 0.01; and ***P < 0.001.

Based on the hypotheses that volume would decrease, and FW increase with increasing depression severity, one-sided Pearson correlations were performed. After Bonferroni correction for 12 comparisons, volume of the right PCC was negatively correlated with GDS scores (*r* = −0.286, *P* = 0.03, BCa 95% CI [−0.55, 0.04]), whereby volume decreased as depression severity increased (see Fig. 3). FW correlated positively with GDS scores in the right PCC (*r* = 0.4, *P* = 0.024, BCa 95% CI [0.17, 0.59]), left (*r* = 0.31, *P* = 0.039, BCa 95% CI [0.11, 0.56]) and right mPFC (*r* = 0.3, *P* = 0.048, BCa 95% CI [0.05, 0.47]), indicating that FW increases with depression severity in the seed regions of the DMN. While no significant main effects or interactions were found with volume, a repeated-measures ANCOVA with FW identified a main effect of *group* [*F*(2,55) = 5.52, *P* = 0.006, *n_p_^2^* = 0.162]. Bonferroni corrected post-hoc tests (nine comparisons) identified increased FW in the D+ group compared to the HC group in the left NAc [*t*(26) = 3.13, *P* = 0.036], right PCC [*t*(26) = 3.24, *P* = 0.039], left [*t*(26) = 2.79, *P* = 0.019] and right mPFC [*t*(26) = 3.46, *P* = 0.027]. Increased FW was also observed in the D+ group compared to the D− group in the right PCC [*t*(42) = 2.98, *P* = 0.014] and left mPFC [*t*(42) = 3.43, *P* = 0.027].

##### White matter microstructure

**Figure 4:**
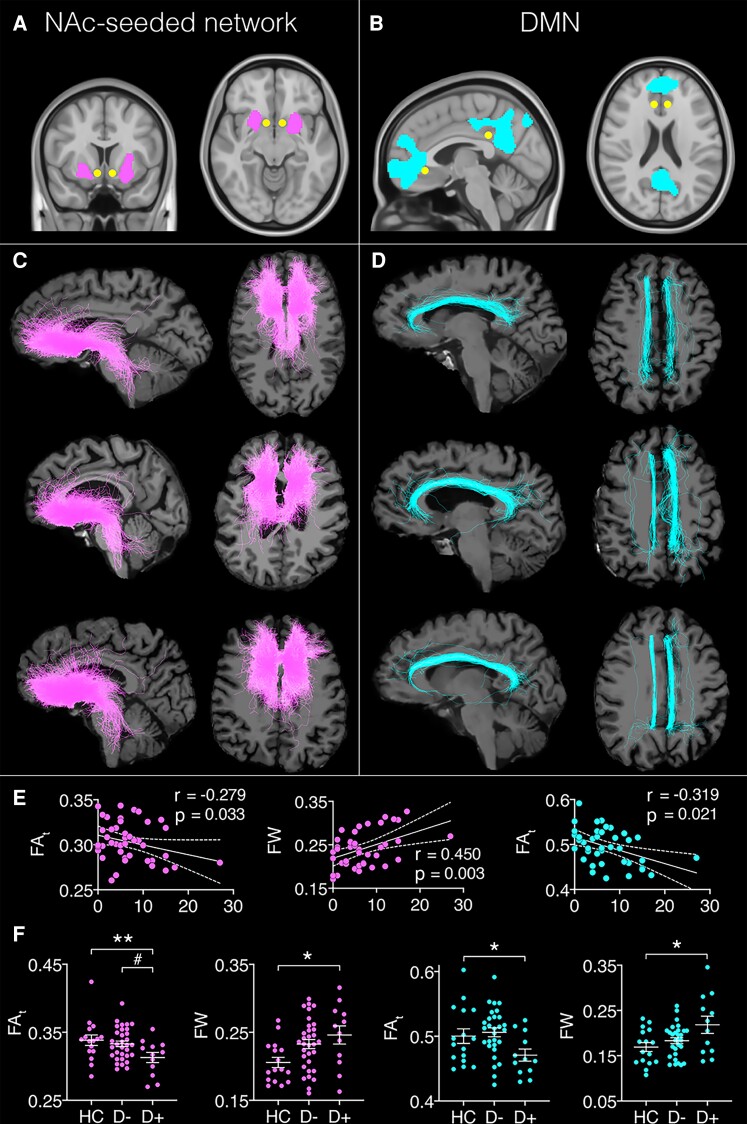
**Regions of interest (ROIs) used for tractography and reconstructed white matter tracts.** Inclusion ROIs are (**A**) nucleus accumbens (NAc) seed (yellow) and cluster with significant BOLD time series correlations associated with geriatric depression scale (GDS) scores (pink); and (**B**) posterior cingulate cortex (PCC) and medial prefrontal cortex (mPFC) seeds (yellow) of the default mode network (DMN) and cluster with significant BOLD time series correlations associated with GDS scores (blue**)**; and (**C**) example of white matter tracts reconstructed for three participants using the ROIs of the NAc-seeded network, warped to individual subject space. The majority of fibres map onto the medial forebrain bundle. (**D**) Example of white matter tracts from three participants using the ROIs of the DMN, warped to individual subject space. The majority of fibres map onto the dorsal cingulum bundle. (**E**) Significant correlations between FA_t_/FW and GDS scores in the medial forebrain bundle (pink) and dorsal cingulum bundle (blue). (**F**) Significant group differences in FA_t_/FW for the medial forebrain bundle (pink) and dorsal cingulum bundle (blue) derived from Bonferroni-corrected post-hoc tests. HC = healthy controls, D+ = stroke patients with depression and D− = stroke patients free of depression. #*P* < 0.075; *P < 0.05; **P < 0.01; and ***P < 0.001.

Tractography analysis suggested that the majority of fibres connecting NAc to the functionally correlated subcortical and frontal lobe cluster coursed in the medial forebrain bundle (MFB). Connection streamlines between ROIs in the DMN were located in the dorsal cingulum bundle (see Fig. 4). FA_t_ was expected to decrease and FW to increase with increasing depression severity. Hence, one-sided Pearson correlations were used. After Bonferroni-correction for four comparisons, FA_t_ correlated negatively with GDS scores in both tracts (MFB: *r* = −0.436, *P* = 0.028, BCa 95% CI [−0.61, −0.23]) and dorsal cingulum bundle: *r* = −0.319, *P* = 0.043, BCa 95% CI [−0.01, 0.42]. In both cases, FA_t_ decreased with increasing depression severity. FW correlated positively (*r* = 0.379, *P* = 0.028, BCa 95% CI [−0.05, 0.65]) with GDS scores in tracts interconnecting the NAc-seeded network (i.e. MFB), whereby FW increased with increasing depression severity. A MANCOVA identified significant main effects for *FA_t_* [MFB: *F*(4,55) = 7.91, *P* = 0.001, *n_p_^2^* = 0.23; dorsal cingulum bundle: *F*(4,55) = 3.63, *P* = 0.033, *n_p_^2^* = 0.119] and *FW* [MFB: *F*(4,55) = 4.26, *P* = 0.019, *n_p_^2^* = 0.139; dorsal cingulum bundle: *F*(4,55) = 4.29, *P* = 0.019, *n_p_^2^* = 0.139)]. Bonferroni corrected post-hoc tests (12 comparisons) revealed that FA_t_ was significantly reduced and FW significantly increased in the D+ group compared to the HC group in fibres of the NAc-seeded network, i.e. the MFB [FA_t_: *t*(26) = 3.97, *P* = 0.012; FW: *t*(26) = 2.84, *P* = 0.019] and tracts interconnecting the DMN, i.e. the dorsal cingulum bundle [FA_t_: *t*(26) = 2.67, *P* = 0.03; FW: *t*(26) = 2.89, *P* = 0.017]. No significant differences in FA_t_ or FW were observed between the D+ and D− groups or the D− and HC groups.

Of note, while apathy was associated with structural measurements (FA_t_ of fibres interconnecting the NAc-seeded network, volume of the right PCC and FW of the right mPFC), none of the correlation coefficients remained significant under the bootstrapped 95% CI and none of the functional estimates were associated with apathy. Furthermore, all main effects remained significant, and no additional effects were observed when removing sex as covariate from the analyses (see Supplements).

### Lesion-network overlap

Lesion overlap with networks, tracts and clusters ranged from 0.02% to 1.28%. No significant group differences or associations with GDS scores were observed for Jaccard indices or voxel overlap with lesions (see Supplementary Fig. 2 and Supplementary Table 2).

## Discussion

Investigation of functional networks in a post-stroke cohort led to three main findings. Depressive symptoms were associated with *hyperconnectivity* in two functional networks: one seeded from NAc, and the DMN. Second, anticorrelation of activity between NAc-seeded network and DMN diminished with increasing depressive symptoms, indicating altered interactions between these networks. In patients reaching criteria for PSD, anticorrelation was lost entirely. Mutual inhibition between task-associated networks and DMN is a characteristic feature of large-scale neural dynamics in the human brain. The current data show that in PSD, this mutual inhibition is lost between a network involved in reward processing and the DMN. Finally, volumetric and microstructural alterations were found within relevant networks suggesting that alterations in functional dynamics reflect pervasive structural change after stroke.

Disturbance of reward processing, through altered function in a network of regions linked to NAc, is a plausible mechanism for the emergence of low mood after stroke. BOLD activity in NAc was positively correlated with activity in a network of regions which subsumed the basal ganglia and limbic system and extended to medial frontal cortex, ACC, and orbitofrontal cortex. To our knowledge, this is the first study to identify enhanced functional activity in a network implicated in reward^32^ to be associated with PSD. Functional aberrations in this network have been reported in MDD and late-life depression, where they have been linked to enhanced reactivity to punishment and increased suicidality.^33^ Our findings therefore suggest that depressive symptoms may share a mechanism of altered reward processing that is common between PSD and other depressive disorders. Previous studies of anticorrelation between DMN and the salience network have posited an inability to shift from self-focused thinking to goal-directed behaviour as a behavioural consequence of reduced network interactions.^16^ An inability to shift to reward-directed behaviour could also contribute to depressive symptoms, particularly behavioural apathy.

A plethora of depression studies,^34^ including studies in PSD,^35^ have provided evidence for aberrant DMN activity. The majority of studies reported increased functional connectivity in the DMN,^36^ yet other studies observed decreased activity in this system.^34^ It is possible that varying activity patterns are linked to different stages of depression, as they often differ across first-episode treatment-naïve,^37^ recurrent,^34^ and remitted depression^38^ samples. It has been suggested that rumination—a key feature of depression—is associated with excessive DMN activity, which in turn is thought to exacerbate switching out of self-reflective states in response to external demands.^39^ A recent meta-analysis of 14 fMRI studies in healthy individuals confirmed that DMN hyperactivity is a principal neural substrate of rumination *specifically*, as opposed to depression *per se*.^40^ In clinical manifestations of depression, rumination is associated with severity, onset and duration of depression.^40^ Collectively, these findings implicate several new treatment strategies for depression. During rumination, areas of the DMN promote the repetitive and passive recall of personally relevant past events. Behavioural interventions focused on mental construction of novel experiences as well as inhibition of mPFC and PCC via transcranial magnetic stimulation could disrupt patients’ cognitive processes associated with rumination^40^ and may represent prophylactic strategies to prevent the development of depression in stroke survivors.

Interestingly, our findings additionally reveal a connection between hyperconnectivity in the DMN and activity in the NAc-seeded network: PSD patients exhibited a reversal of anticorrelation between these two networks. During externally, goal-directed attention, DMN activity reduces in a load-dependent fashion as cognitive demands increase^41^ such that activity in the DMN decreases with increasing activity in NAc-seeded reward circuits. Greater anticorrelation between the two systems is linked to more efficient value-guided behaviour and reward attitudes in humans,^42^ which indicates that coupling strength of the two networks is an important factor in successful application of reward processing in goal-directed behaviour. NAc plays a central role in the pathophysiology of reward deficits and inefficient interactions with the DMN are thought to be implicated in the manifestation of anhedonia^43^ across mood and psychotic disorders. A study by Sharma *et al*.^44^ investigated reward responsivity in patients with MDD,^44^ bipolar disorder, schizophrenia, and healthy controls. The study observed decoupling between NAc and DMN regions with increasing reward sensitivity, and hyperconnectivity within the DMN, to be associated with reward deficits across all clinical groups. This lack of integration between DMN and NAc activity was interpreted to reflect a brain phenotype of impaired reward-oriented internal cognition, expressed externally as anhedonia. A failure of efficient communication between these networks may therefore represent a cross-diagnostic neurological signature of reward-related depressive symptoms.

Impaired interaction between reward-processing and DMN in the brain provides a plausible causal mechanism for the emergence of depressive symptoms after stroke. Such an account would also provide an explanation for the absence of a simple consistent locus for depression in lesion-mapping studies. Correlation of BOLD signal variation suggests that NAc is functionally interconnected with multiple subcortical structures as well as regions of medial frontal and temporal cortex. Similarly, the DMN spans a distributed network of regions in frontal and parietal cortices. Consequently, structural lesions in a wide range of locations could potentially disturb both within-network and between-network functional interactions. While there is conflicting evidence as to whether left or right hemisphere lesions are more strongly associated with the development of post-stroke depression,^3^ it is possible that lesions in either hemisphere have differential effects on bilateral functional network activity.

Microstructure of the medial forebrain bundle was associated with GDS scores. The medial forebrain bundle is implicated in reward processing through its connectivity with structures known to be critical for reward and through carriage of the mesolimbic dopaminergic pathway from the ventral tegmental area to NAc.^45^ Furthermore, axonal tracing studies in rodents and non-human primates have shown that it carries direct connections to mPFC.^46,47^ It is therefore a candidate pathway for functional interactions between NAc, regions of the mPFC involved in reward processing, and potentially other networks that involve mPFC including the DMN. Consistent with this account, microstructure of mPFC regions correlated with depressive symptoms, in a way that paralleled the associations found for microstructure of the medial forebrain bundle.

Structural changes in conjunction with hyperconnectivity were also found within the DMN. Lower grey matter volume in the PCC was associated with higher depression scores. Volume reduction in PCC has previously been reported in patients with MDD and has been linked to altered DMN activity.^13^ The PCC is one of the most metabolically active brain regions and has dense structural connections to widespread regions of the brain.^48^ As part of the DMN, the PCC is most active during self-referential processes, but is simultaneously the main cortical hub responsible for switching between DMN and other network activity in response to environmental changes requiring behavioural adaptation.^48^ It is possible that morphometric changes of PCC play a role in the loss of efficient cross-network communication between DMN and the NAc-seeded reward network, in patients with PSD. Microstructure of the dorsal cingulum bundle, which contains projections connecting PCC and precuneus to medial prefrontal cortex in the DMN,^49,50^ also exhibited microstructural properties associated with depression severity in stroke survivors.

The basis for the structural alterations associated with depressive symptoms in some individuals remains unclear. The brain undergoes dynamic changes in the weeks to months after stroke, which extend beyond the initial site of injury. Microglial activation, for example, is known to propagate along white matter pathways away from the site of infarction,^51,52^ and elevation of extracellular free water—as demonstrated in the medial forebrain bundle and dorsal cingulum in this study—has been considered a putative marker of neuroinflammation. This account, however, remains speculative. The main alternative explanation is that a structural pattern of vulnerability to depressive symptoms is present prior to stroke and that the interaction between stroke and pre-existing vulnerability precipitates the emergence of depressive symptoms.

As expected, no association was found between functional connectivity in the control network seeded from V1 and depressive symptoms in PSD. However, contrary to our predictions, a lack of association between BOLD activity and depression severity was also observed in amygdala and dlPFC-seeded networks. Functional changes in amygdala-seeded structures have previously been linked to increased cortisol levels in patients with MDD and patients with bipolar disorder who experienced depressive episodes.^7^ Cortisol levels are elevated in the first few days to weeks after stroke^53^ which may explain why studies by Zhang *et al*.^14,54^ observed hyperconnectivity in this network in patients with PSD within the first 10 days of stroke. The current study was conducted approximately three months after stroke, when cortisol levels are typically restored to the normal reference range.^53^ Aberrant signalling in cortico-cortical circuits are commonly observed in healthy individuals with a family history of depression^55^ and in patients with MDD who achieve remission from depressive symptoms but continue to experience cognitive impairments.^39^ It is possible that depression experienced during different stages of recovery may be caused by different underlying neural mechanisms.

Recent years have seen increasing concerns regarding the reproducibility of neuroimaging findings and the lack of adequately powered studies needed to identify reliable measurements within individual samples.^56^ In order to provide an additional layer of confidence in our findings, we supplemented brain-depression correlates with additional bootstrapped confidence intervals. Pearson correlations between GDS scores and measurements of PCC volume, FA_t_ and FW in the dorsal cingulum did not remain significant under the bootstrapped confidence intervals. Bootstrapping has been reported to improve the reliability of single study research.^57^ As such, the correlations between these structural correlates and depression severity should be treated with caution until replication in better-powered studies is possible. Of note, due to its high dimensionality, uncertainty in test–retest reliability has been particularly strong for estimates of functional connectivity.^58^ Attempts to replicate many fMRI experiments have failed, which is further complicated by the variability in acquisition parameters and analysis pipelines used across studies. Ultimately, large brain-imaging efforts, such as the UK Biobank, and standardized processing pipelines are needed to provide confidence in the findings of smaller neuroimaging studies,^58^ such as the study presented here. Nevertheless, in the present study, confidence in the correlations between functional connectivity and depression severity were strengthened by the finding that all bootstrapped confidence intervals remained significant, lending a degree of confidence in our findings until replication in larger samples is possible. Despite the adverse consequences of post-stroke depression on functional recovery and quality of life,^1^ estimates of depression from multi-ethnic, diverse stroke samples is not readily available. Routine collection of depression measures in neuroimaging stroke studies, clinical trials and observational stroke studies in general would enable the replication of imaging findings in larger, multi-ethnics stroke samples and expedite the identification of reliable risk-factors and crucial time periods for intervention that could ultimately limit the progression to florid depression in stroke survivors.

Cerebrovascular pathology is often found to coexist with late-life depression so that the boundaries of these two disorders converge in older people. There is a need for future studies to investigate etiological brain-based mechanisms of depression across varying ages and in the context of neurological and vascular disorders. Individual neuropsychiatric symptoms may exhibit dissociable patterns of association with the observed brain aberrations. While we tested associations between brain measurements and apathy items from the GDS, future studies may wish to explore a wider breadth of depression symptomatology and comorbid psychiatric complications. Methodological limitations of the current study include the restriction of the diffusion MRI acquisition to a single shell so that more advanced compartment models could not be performed.

Collectively, the findings from this study suggest that aberrant activity within a network involving NAc, specialized for processing signals linked to reward, and interactions between this network and the DMN may give rise to depressive symptoms after stroke. Longitudinal studies are now needed to track the emergence and persistence of these structural and functional features after stroke. Use of other imaging modalities, blood and CSF biomarkers will help to elucidate the nature of these changes and investigate a putative role for neuroinflammation, similar to that proposed for MDD in the absence of stroke.

## Supplementary Material

fcac281_Supplementary_Data

## Abbreviations

ANCOVA =: analysis of covariances
ACC =: anterior cingulate cortex
BCa =: bias-corrected and accelerated
BOLD =: blood oxygen level dependent
CI =: confidence interval
dACC =: dorsal anterior cingulate cortex
dlPFC =: dorsolateral prefronal cortex
D + =: group of patients with post-stroke depression
D− =: group of patients free of post-stroke depression
DMN =: default mode network
DSM-IV =: Diagnostic and statistical; manual of mental of mental disorder, fourth edition
EPI =: echo-planar imaging
FAt =: tissue-specific fractional anisotropy
FC =: functional connectivity
FLAIR =: fluid-attenuated inversion recovery
fMRI =: functional magnetic resonance imaging
FRFSE =: fast recovery fast spin echo
FOD =: fibre orientation distributions
FOV =: field of view
FW =: free-water
FWE =: family-wise error
GDS =: geriatric depression scale
HC =: healthy controls
iFOD2 =: 2nd order integration over fibre orientation distribution
M =: mean
MANCOVA =: multivariate analysis of covariances
MDD =: major depressive disorder
MFB =: medial forebrain bundle
MNI =: Montreal Neurological Institute
mPFC =: medial prefrontal cortex
MPRAGE =: magnetization-prepared rapid acquisition gradient echo
NAc =: nucleus accumbens
PCC =: posterior cingulate cortex
PSD =: post-stroke depression
REC =: Research Ethics Committee
rs-fMRI =: resting-state functional magnetic resonance imaging
SD =: standard deviation
SIFT =: spherical deconvolution informed filtering of tractograms
SPM =: statistical parametric mapping
3T =: 3 Tesla
TE =: echo time
TR =: repetition time
V1 =: primary visual cortex
