# Supplementary material for: Hyperconnectivity and altered interactions of a nucleus accumbens network in post-stroke depression
Source: Brain Commun. 2022 Nov 2;4(6):fcac281. doi: 10.1093/braincomms/fcac281 (PMC9677459; doi:10.1093/braincomms/fcac281)
Supplement: fcac281_Supplementary_Data [file fcac281_supplementary_data.pdf]

Supplementary Material

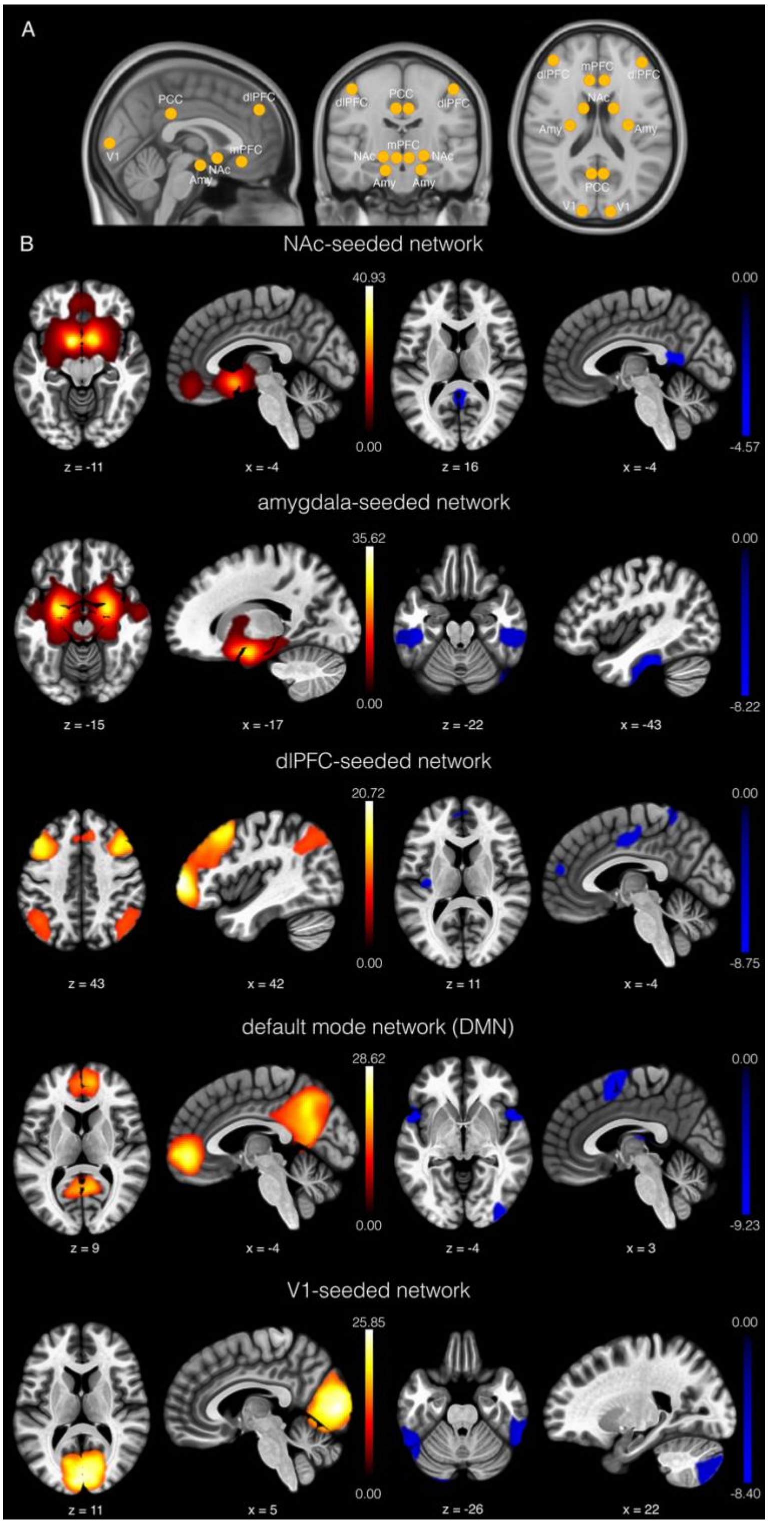

Supplementary Fig. 1. A) Seed regions used to generate five resting state networks: nucleus accumbens (NAc)- seeded network, amygdala (Amy)-seeded network, dorsolateral prefrontal cortex (dlPFC)- seeded network, default mode network (DMN) seeded from posterior cingulate cortex (PCC) and medial prefrontal cortex (mPFC), primary visual cortex (V1)- seeded network. B) Network clusters exhibiting significant BOLD time series correlations with seed regions of the five resting-state networks. Images were FWE-corrected at a cluster threshold of $p < 0.05$. Warm colors (red to yellow) represent positively correlated clusters and cold colors (blue to black) represent anticorrelated clusters. Colorbars represent t-statistic values.

Associations between apathy and brain function and structure measurements

Factor analyses of the GDS have identified a cluster including the items "dropped activities and interests", "prefer to stay at home" and "lack of energy", which has since been suggested as the 'Withdrawal-Apathy-Lack of Vigor' factor. Several studies have referred to this factor as the apathy sub-scale of the GDS and it has been confirmed to have moderate accuracy at identifying apathy when compared to the Apathy Scale.[1] We therefore repeated all Pearson correlations performed between the total GDS score and measures of brain function or structure with an apathy score derived from these three GDS items.

Apathy was negatively correlated with FA_t in the medial forebrain bundle MFB (r=-0.286, p=0.031, BCa 95% CI [-0.56, 0.06]), which interconnects the NAc-seeded network and volume of the right PCC (r=-0.286, p=0.031, BCa 95% CI [-0.49, 0.07]). Apathy was positively correlated with free-water in the right mPFC (r=0.288, p=0.031, BCa 95% CI [0.03, 0.57]).

Analysis of main effects without sex as covariate

Associations between PSD and resting-state networks

A repeated-measures ANCOVA with the between-subject factor *group* (HC/D-/D+) and the within-subjects factors *network* (NAc-seed network/ DMN) and *cluster* (cluster 1/ cluster 2) identified a significant main effect of *group* ($F(2,56)=6.477$, $p=0.003$, $n_p^2=0.191$).

Anticorrelations between DMN and NAc-seeded network

A MANCOVA identified main effects of *group* for FC between bilateral NAc and PCC/precuneus ($F(3,56)=7.82$, $p< 0.001$, $n_p^2=0.295$), PCC and thalamus ($F(3,56)=6.84$, $p<0.001$, $n_p^2=0.268$) and mPFC and thalamus ($F(3,56)=3.66$, $p=0.018$, $n_p^2=0.164$).

Grey matter volume and microstructure

While no significant main effects or interactions were found with volume, a repeated-measures ANCOVA with FW identified a main effect of *group* ($F(2,56)=6.38$, $p=0.003$, $n_p^2=0.186$).

White matter microstructure

A MANCOVA identified significant main effects for FA_t (MFB: $F(3,56)=7.37$, $p=0.009$, $n_p^2=0.12$; dorsal cingulum bundle: $F(3,56)=4.04$, $p=0.049$, $n_p^2=0.067$) and *FW* (MFB: $F(3,56)=3.09$, $p=0.034$, $n_p^2=0.142$; dorsal cingulum bundle: ($F(3,56)=3.88$, $p=0.014$, $n_p^2=0.172$).

**Supplementary Table 1.** Clusters correlated/anticorrelated with seed-regions			
	max intensity voxel	cluster size (voxels)	cluster location (number of voxels)
NAc-seeded network – correlations			
cluster 1	–26 +28 +00	13 767	left orbitofrontal cortex (1000)<br>ventral anterior cingulate cortex (910)<br>right orbitofrontal cortex (881)<br>medial frontal cortex (689)<br>left putamen (575)<br>right putamen (476)<br>left insular (370)<br>left amygdala (321)<br>right amygdala (291)<br>left dorsal anterior cingulate cortex (280)<br>right insular cortex (260)<br>left caudate (258)<br>right caudate (230)<br>left pallidum (161)<br>right dorsal anterior cingulate cortex (147)<br>left thalamus (146)<br>right thalamus (126)<br>right pallidum (124)<br>right hippocampus (106)<br>left hippocampus (75)<br>anterior cingulate gyrus (64)
NAc-seeded network – anticorrelations			
cluster 1	+02 –50 +06	820	posterior cingulate cortex (358)<br>precuneous (244)
Amy-seeded network – correlations			
cluster 1	–26 –06 –20	12 519	right putamen (684)<br>left putamen (656)<br>left hippocampus (639)<br>right parahippocampal gyrus (603)<br>middle temporal gyrus (600)<br>right hippocampus (562)<br>left parahippocampal gyrus (554)<br>right orbitofrontal cortex (447)<br>left orbitofrontal cortex (421)<br>left temporal pole (314)<br>right temporal pole (300)<br>right superior temporal gyrus (263)<br>right insular (226)<br>left insular (222)<br>ventral anterior cingulate cortex (109)

			left pallidum (106)<br>right pallidum (103)<br>left planum polare (69)<br>right planum polare (55)<br>left superior temporal gyrus (51)<br>left nucleus accumbens (40)<br>right nucleus accumbens (24)<br>right lingual gyrus (21)<br>left thalamus (11)<br>left lingual gyrus (2)
Amy-seeded network – anticorrelations			
cluster 1	+38 –26 –30	683	right inferior temporal gyrus (386)<br>right temporal fusiform gyrus (142)
cluster 2	–44 –30 –26	659	left inferior temporal gyrus (318)<br>left temporal fusiform gyrus (133)
dlPFC-seeded network – correlations			
cluster 1	–46 +26 +22	5 902	left middle frontal gyrus (2181)<br>left frontal pole (2052)<br>left inferior frontal gyrus (663)<br>left superior frontal gyrus (169)<br>left orbitofrontal gyrus (21)
cluster 2	+42 +58 +02	4 912	right frontal pole (2220)<br>right middle frontal gyrus (1812)<br>right inferior frontal gyrus (158)<br>right superior frontal gyrus (91)
cluster 3	+46 –50 +36	1 104	right lateral occipital cortex (532)<br>right angular gyrus (477)<br>right supramarginal gyrus (13)
cluster 4	–44 –60 +44	944	left lateral occipital cortex (498)<br>left angular gyrus (229)<br>left supramarginal gyrus (46)<br>left superior parietal lobule (5)
cluster 5	+10 +30 +44	170	right dorsal anterior cingulate cortex (32)<br>right superior frontal gyrus (30)<br>left superior frontal gyrus (30)<br>left dorsal anterior cingulate cortex (16)
dlPFC-seeded network – anticorrelations			
cluster 1	–22 –40 +76	2 946	left postcentral gyrus (646)<br>right superior parietal lobule (480)<br>right postcentral gyrus (404)<br>precuneous (385)<br>left superior parietal lobule (267)

			left precentral gyrus (131)<br>left superior frontal gyrus (30)<br>right lateral occipital cortex (9)
cluster 2	–06 –02 +44	730	anterior cingulate gyrus (327)<br>left supplementary motor area (158)<br>left precentral gyrus (55)<br>right supplementary motor area (39)<br>right precentral gyrus (37)<br>posterior cingulate cortex (14)
cluster 3	–36 –16 +10	301	left insular cortex (169)<br>left central opercular cortex (41)<br>left Heschl's gyrus (33)<br>left parietal operculum cortex (18)<br>left putamen (1)
cluster 4	+00 +56 +18	245	left anterior cingulate cortex (60)<br>right frontal pole (43)<br>right anterior cingulate cortex (29)<br>left frontal pole (26)<br>left superior frontal gyrus (23)<br>right superior frontal gyrus (16)
cluster 5	+26 –06 –24	234	right hippocampus (93)<br>right amygdala (70)<br>right parahippocampal gyrus (54)
DMN – correlations			
cluster 1	+06 –56 +40	7 692	precuneous (4235)<br>posterior cingulate cortex (1561)<br>lingual gyrus (286)
cluster 2	+06 +60 –04	4 332	frontal poles (1286)<br>dorsal anterior cingulate gyri (1050)<br>medial frontal cortex (512)<br>anterior cingulate gyrus (422)<br>superior frontal gyrus (8)
cluster 3	+46 –68 +26	862	right angular gyrus (629)<br>right middle temporal gyrus (4)
cluster 4	–42 –78 +30	614	left angular gyrus (364)
DMN - anticorrelations			
cluster 1	–46 –60 –30	2 251	left cerebellum (1658)<br>left occipital fusiform gyrus (115)<br>left occipital pole (114)<br>left temporal occipital fusiform cortex (40)<br>left lateral occipital cortex (33)<br>left inferior temporal gyrus (31)<br>left lingual gyrus (9)
cluster 2	+46 +08 +22	1 628	right precentral gyrus (597)<br>right inferior frontal gyrus (251)

			right temporal pole (205)<br>right opercular cortex (130)<br>right middle frontal gyrus (63)<br>right insular (51)<br>right orbitofrontal cortex (14)<br>right planum polare (14)
cluster 3	+38 –86 –04	1 599	right cerebellum (903)<br>right lateral occipital cortex (397)<br>right occipital pole (163)<br>right occipital fusiform gyrus (7)
cluster 4	–52 +10 –02	1 364	left precentral gyrus (495)<br>left inferior frontal gyrus (257)<br>left temporal pole (184)<br>left central opercular cortex (90)<br>left frontal operculum cortex (44)<br>left middle frontal gyrus (22)<br>left insular (9)<br>left inferior frontal gyrus (9)<br>left planum polare (8)<br>left orbitofrontal cortex (4)
cluster 5	+04 +04 +62	1 020	supplementary motor cortex (477)<br>superior frontal gyrus (177)<br>right dorsal anterior cingulate cortex (125)
cluster 6	+56 –40 +54	426	right posterior supramarginal gyrus (263)<br>right anterior supramarginal gyrus (76)<br>right superior parietal lobule (19)<br>right angular gyrus (2)
cluster 7	+00 –26 +10	112	left thalamus (35)<br>right thalamus (13)
V1-seeded network – correlations			
cluster 1	+02 –86 +06	19 801	right lingual gyrus (1684)<br>right occipital pole (1586)<br>precuneous (1577)<br>left occipital pole (1568)<br>left lingual gyrus (1439)<br>right occipital fusiform cortex (919)<br>right calcarine cortex (894)<br>left occipital fusiform cortex (791)<br>left calcarine cortex (706)<br>right lateral occipital cortex (652)<br>right cuneal cortex (641)<br>left lateral occipital cortex (575)<br>posterior cingulate gyrus (129)<br>left parahippocampal gyrus (44)<br>right parahippocampal gyrus (8)

V1-seeded network – anticorrelations			
cluster 1	+36 –74 –56	9 896	cerebellum (4137)<br>right interior temporal gyrus (948)<br>left inferior temporal gyrus (925)<br>left middle temporal gyrus (446)<br>right middle temporal gyrus (285)

Note. Number of voxels per region do not add up to total cluster size due to voxels not covered by atlas labels. NAc = nucleus accumbens; Amy = amygdala; dlPFC = dorsolateral prefrontal cortex; DMN = default mode network; V1 = primary visual cortex; All clusters are significant at a FWE-corrected alpha level of $p < 0.05$.

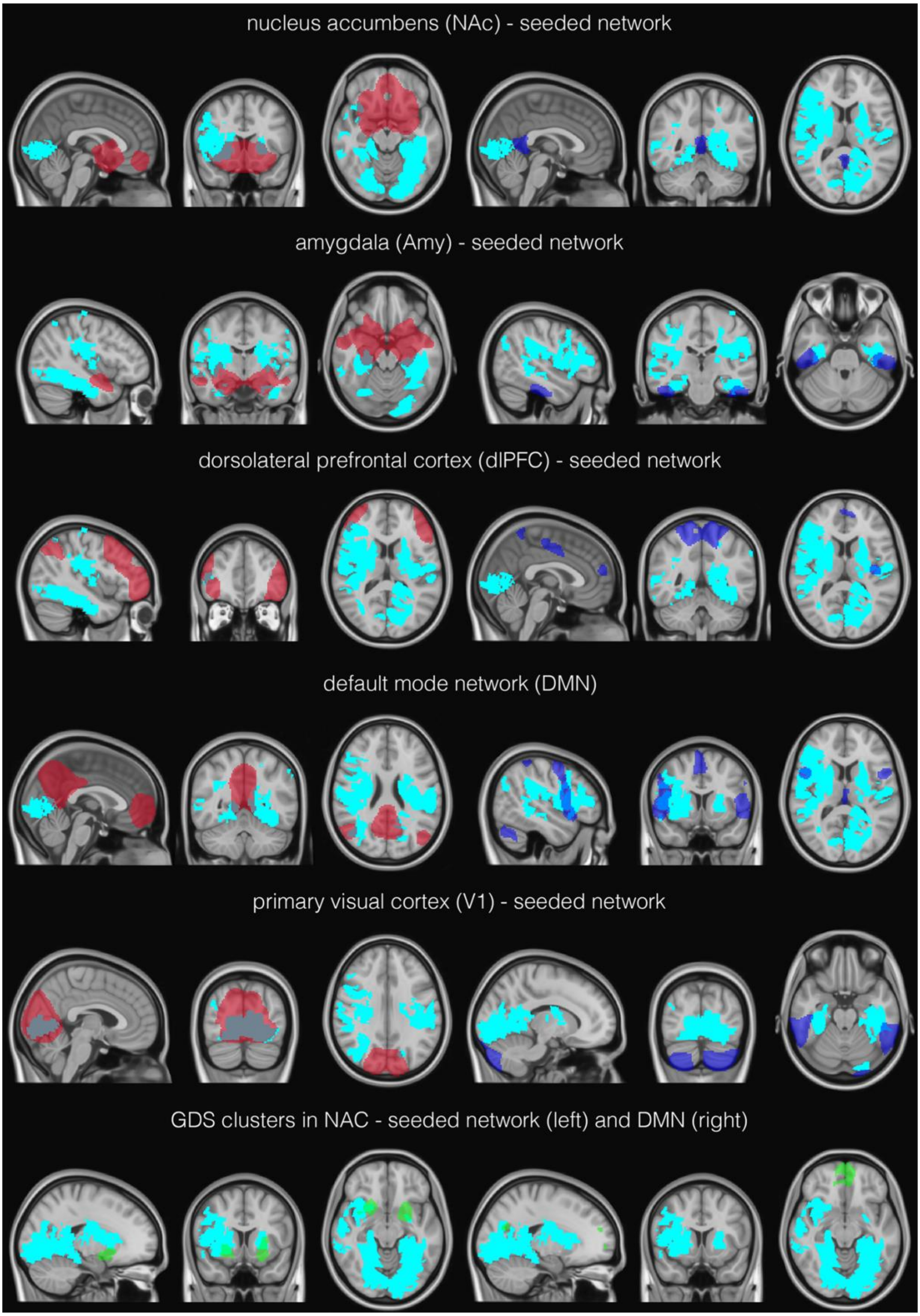

Supplementary Fig. 2. Lesions from 44 stroke patients (light blue) were overlaid on resting-state functional connectivity networks that were correlated (red) anticorrelated (blue) and associated with GDS scores (green) with seed regions.

Supplementary Table 2. Lesion overlap with FC networks, clusters associated with GDS scores, and white matter tracts

		jaccard index				voxels			
	n	mean	*SD*	min	max	mean	*SD*	min	max
NAc-seeded									
correlated	16	0.0063	0.0155	0	0.071	534.57	1339.2	0	6440
anticorrelated	3	0.0002	0.0009	0	0.005	6.52	28.13	0	170
GDS cluster	12	0.0104	0.027	0	0.107	236.11	620.834	0	2389
WM tracts	6	0.0002	0.0008	0	0.004	2.17	7.68	0	43
Amy-seeded									
correlated	24	0.0078	0.0132	0	0.051	909.5	1527.07	0	6078
anticorrelated	4	0.0021	0.0078	0	0.045	104.25	400.04	0	2397
dlPFC-seeded									
correlated	10	0.0011	0.0035	0	0.016	327.73	1009.26	0	4469
anticorrelated	9	0.001	0.004	0	0.025	59.41	254.76	0	1655
DMN									
correlated	10	0.002	0.0059	0	0.027	588.32	1711.6	0	8317
anticorrelated	11	0.0023	0.0072	0	0.038	259.05	845.36	0	4783
GDS cluster	4	0.0003	0.0013	0	0.007	31.75	151.33	0	868
WM tracts	4	0.0004	0.0009	0	0.005	1.58	3.9	0	21
V1-seeded									
correlated	8	0.0128	0.0365	0	0.174	2380.89	6876.92	0	33397
anticorrelated	6	0.001	0.0035	0	0.019	126.82	453.49	0	2592

Note. FC = functional connectivity; GDS = geriatric depression scale; SD = standard deviation; NAc = nucleus accumbens; Amy = amygdala; dlPFC = dorsolateral prefrontal-cortex; DMN = default mode network; V1 = primary auditory cortex.

References

1. Szymkowicz SM, Ellis LJ, May PE. The 3-Item “Apathy” Subscale Within the GDS-15 Is Not Supported in De Novo Parkinson’s Disease Patients: Analysis of the PPMI Cohort. *J Geriatr Psychiatry Neurol*. 2021;35(3):309-316.
